# Gas Chromatography–Mass Spectrometry-Based Analyses of Fecal Short-Chain Fatty Acids (SCFAs): A Summary Review and Own Experience

**DOI:** 10.3390/biomedicines12081904

**Published:** 2024-08-20

**Authors:** Paweł Czarnowski, Michał Mikula, Jerzy Ostrowski, Natalia Żeber-Lubecka

**Affiliations:** 1 Department of Genetics, Maria Sklodowska-Curie National Research Institute of Oncology, 02-781 Warsaw, Poland; michal.mikula@nio.gov.pl (M.M.); jostrow@warman.com.pl (J.O.); natalia.zeber-lubecka@cmkp.edu.pl (N.Ż.-L.); 2 Department of Biochemistry, Radioimmunology and Experimental Medicine, Children’s Memorial Health Institute, 04-736 Warsaw, Poland; 3 Department of Gastroenterology, Hepatology and Clinical Oncology, Centre of Postgraduate Medical Education, 01-813 Warsaw, Poland

**Keywords:** SCFA, GC/MS, stool metabolites

## Abstract

The gut microbiome, crucial to human health, changes with age and disease, and influences metabolic profiles. Gut bacteria produce short-chain fatty acids (SCFAs), essential for maintaining homeostasis and modulating inflammation. Dysbiosis, commonly due to poor diet or lifestyle, disrupts the integrity of the intestinal barrier and may contribute to conditions such as obesity, diabetes, and non-alcoholic fatty liver disease (NAFLD). Analytical methods such as gas chromatography–mass spectrometry (GC/MS) are vital for SCFA analysis, with various preparation and storage techniques improving the accuracy. Advances in these methods have improved the reliability and sensitivity of SCFA quantification, which is crucial for the identification of disease biomarkers. Evidence from GC/MS-based studies has revealed that accurate SCFA quantification requires meticulous sample preparation and handling. The process begins with the extraction of SCFAs from biological samples using methods such as direct solvent extraction or solid-phase microextraction (SPME), both of which require optimization for maximum recovery. Derivatization, which chemically modifies SCFAs to enhance volatility and detectability, is a crucial step, typically involving esterification or silylation. Following this, the cleanup process removes impurities that might interfere with the analysis. Although recent advances in GC/MS technology have significantly improved SCFA-detection sensitivity and specificity, proper sample storage, with acid preservatives and the avoidance of repeated thawing, is essential for maintaining SCFA integrity.

## 1. Introduction

Multi-omics is a method of analysis that allows for the combining and analyzing of data obtained from two or more omics methods (e.g., metagenomics, metabolomics, or transcriptomics) to search for and understand the mechanisms of biological processes leading to the development of metabolic diseases in humans [1]. The central tenet is that multi-omics data may help us track related molecular changes at different biological levels with genetic variants [2]. A large amount of data received allows for better interpretation and visualization of the results obtained from the same biological sample [3]. The data may also be used to look for relationships between the host and its inner microbiome, the composition of which changes with age. The development of metabolic diseases also leads to a change in the composition of the microbiota, which translates into changes in its metabolomic profile. Continuous development of analytical methods allows for the determination of an increasing number of metabolites produced by bacteria, the impact of which on the functioning of the human body has not been fully determined yet. Gut bacteria are the largest group of micro-organisms living in the human gastrointestinal tract. The gut microbiome (GM) contains about 10^15^ microbial cells and over 22 million microbial genes, both of which exceed the number of cells and genes in a human [4]. Such a large number of genes allows bacteria to produce a wide range of metabolites with various biological activities that perform very important roles, ranging from the synthesis of vitamin K and group B vitamins, short-chain fatty acids, amino acids (AAs) and secondary bile acids to anti-inflammatory mediators and many more. An analysis of human gut bacteria for B vitamin biosynthesis pathways showed that 40 to 65% of all bacteria were capable of synthesizing all eight B vitamins [5]. The intestinal microbiome can also synthesize phenylalanine and a tyrosine derivative, i.e., dopamine, which may be converted into norepinephrine and epinephrine. The role of the microbiome is crucial in maintaining the human body in homeostasis due to the production of signaling metabolites. In addition, gut bacteria secrete mucins that prevent the colonization of the gut lumen by harmful bacteria which may produce hazardous compounds [6]. The depletion of gut microbiome may result from various factors, such as an unhealthy, Western-style diet, full of processed food rich in carbohydrates and high amounts of fat, lack of movement caused by a sedentary lifestyle, or antibiotic intake which contributes to the occurrence of dysbiosis, characterized by an abnormal change in the composition of gut microbiota on the intestinal surface caused by reduced beneficial bacterial species and increased gut permeability. Various materials may be used for testing: blood, plasma, saliva, cerebrospinal fluid, tissues and urine, with stool being one of the least studied [7]. Stool consists of undigested food remains, water, live and dead bacteria, as well as small and large particles resulting from the digestion of food by enzymes and bacteria. These compounds are then absorbed through the gastrointestinal tract (GIT), where, as it turns out, they play a key role in the proper functioning of the human body. These compounds are widely known as stool metabolome, the analysis of which may provide important information about its composition, although it is different in each of us. This is crucial for searching for biomarkers of diseases and for trying to understand the origin of conditions. The stool is easily available and has great potential as a diagnostic material [8], having an added value not only in the diagnosis of intestinal and rectal diseases. Conversely, feces are a heterogeneous and complex material containing different macro- and microcompounds found among undigested food remains. The variety of food consumed by humans affects the composition and levels of these compounds. Therefore, stool can be a challenging material for analysis [7]. Changes in the levels of individual metabolites do not necessarily correlate with the severity of the disease, so further research and the search for potential biomarkers are necessary. This is why this review is focused on showing the most current information about the methods of the analysis of short-chain fatty acids by gas chromatography (GC) coupled with mass spectrometry (MS).

## 2. The Role of Short-Chain Fatty Acids in the Human Body

**Figure 1:**
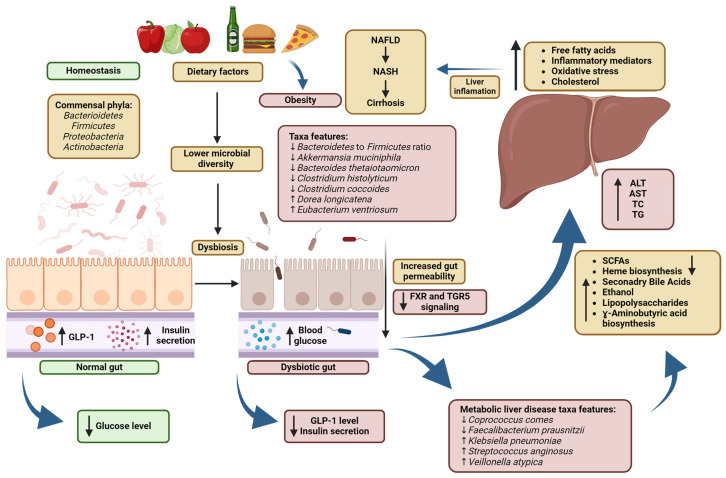
Gut–microbiome relationship. Higher consumption of processed food lowers microbial α-diversity, characteristic of dysbiosis, and increases the intake of processed food. SCFAs (acetate, propionate, butyrate) are produced by fermenting dietary fiber and resistant starch. Key SCFA producers include *Clostridium*, *Roseburia* and *Faecalibacterium*. SCFAs regulate appetite, energy homeostasis and the integrity of the intestinal barrier, altering mineral bioavailability and the metabolism of glucose, lipids and cholesterol. Dysbiosis increases intestinal permeability, allowing harmful compounds to enter the bloodstream, disrupting the gut–liver axis and immune response, and altered SCFA levels contribute to conditions such as obesity, IBS, IBD, colon cancer, celiac disease and NAFLD. Created with BioRender.com (accessed on 17 July 2024).

Short-chain fatty acids (SCFAs) constitute a group of metabolites that is very often measured in the search for potential biomarkers. SCFAs are the largest group of metabolites produced by intestinal bacteria in the process of anaerobic fermentation. Intestinal colonization begins soon after birth, and its course is influenced by environmental factors such as the mode of delivery, feeding, or the use of antibiotics [9]. Research has indicated significant differences in the composition of the microbiota of newborns compared to adults, and its diversity and role have been found to increase most intensively during the first years of life [10]. During the first two years of life, three stages of SCFA profile change may be distinguished. The early phase is characterized by low levels of acetic acid and high levels of succinic acid. The intermediate phase is characterized by high levels of lactic and formic acid, while a high concentration of propionate and butyrate occurs in the late phase [9]. The delay or formation of a different microflora composition in infants is associated with obesity [11]. The loss of *Bifidobacterium* bacteria, which is the most abundant gut bacteria until weaning, was observed to be associated with decreased autoimmune activity and the possible development of allergic disease. The reduction of other early-life bacteria such as *Faecalibacterium*, *Lachnospira*, *Veillonella* and *Rothia,* and the resultant intestinal microbial dysbiosis, have been associated with a higher risk of developing asthma [12,13] and the development of type 1 diabetes, which was correlated with lower amounts of genes for carbohydrate fermentation and SCFA production [14]. After solid food introduction, the gut microbiota starts to develop at a faster pace and begins to resemble the microbiome of an adult person, but still the domination of *Firmicutes* and *Bacteroidetes* phyla is observed in the first two or three years of life, when they make up most of the gut microbiome [15]. Even then, the composition of gut bacteria changes from childhood through adolescence. The increased consumption of processed meat at that age is related to lower microbial α-diversity, characteristic of dysbiosis, and also to a greater intake of processed foods (Figure 1). Furthermore, skipping breakfast is associated with the reduced abundance of potentially beneficial taxa known to produce SCFAs [16]. SCFAs with straight chains (acetate, propionate and butyrate) are produced by the fermentation of dietary fiber and resistant starch in the intestinal lumen [17]. Butyrate is mainly produced by the following genera: *Clostridium*, *Eubacterium* and *Fusobacterium*, but *Clostridium leptum*, *Roseburia* spp., *Faecalibacterium prausnitzii* and *Coprococcus* spp. are the most productive. Propionate is a metabolite of *Bacteroidetes* and *Propionibacterium* [15]. Branched-chain SCFAs (isobutyric acid, isovaleric acid and 2-methylbutyric acid) are produced from branched-chain amino acids (leucine, isoleucine and valine). These compounds are absorbed into the systemic circulation by passive diffusion and active transport, where they influence appetite regulation by binding to the G-protein-coupled free fatty acid receptor 3 (FFAR3) and stimulate leptin secretion by the adipose tissue [18]. Thus, they take part in the maintenance of energy homeostasis.

**Figure 2:**
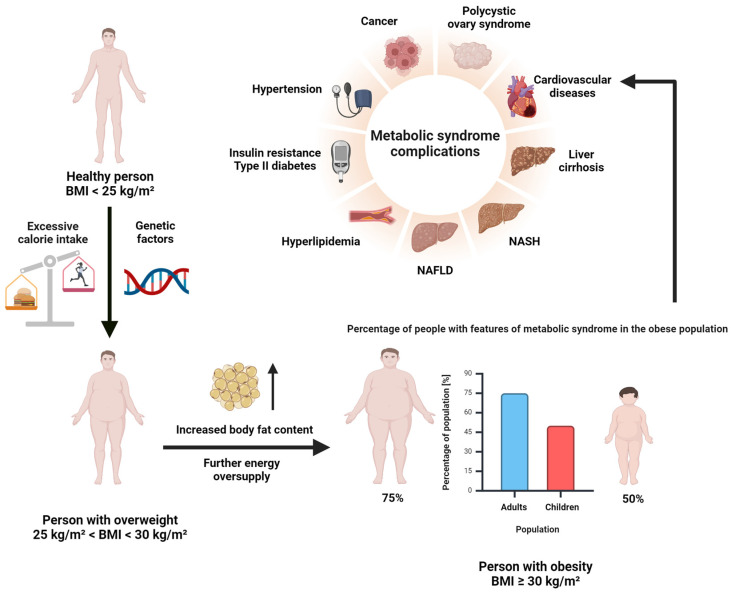
Development of metabolic syndromes through obesity. The prevalence of obesity and related metabolic disorders is increasing due to unhealthy lifestyles, lack of exercise and an excessive intake of empty calories. This causes insulin resistance and may lead to type 2 diabetes. The WHO estimates that 1 in 8 people worldwide is obese, with adult obesity more than doubling since 1990. Up to 75% of obese adults and 50% of obese children develop metabolic disorders. Created with BioRender.com (accessed on 15 July 2024).

SCFAs produced by gut bacteria can affect the bioavailability of minerals due to lumen acidification and changes in the amount of transport proteins on the intestinal surface [19]. The gut microbiome produces tryptophan, which binds to the aryl hydrocarbon receptor (AhR) and enhances the function of the intestinal epithelial barrier, as well as regulatory immune responses of the human body [6]. Butyric acid inhibits histone deacetylases and it is also the main source of energy for colonocytes. Short-chain fatty acids, especially butyrate, can modulate the expression of genes responsible for the synthesis of tight-junction proteins. They also regulate occludin redistribution to prevent increased intestinal permeability [4]. Acetic acid is the most abundant SCFA in both intestinal lumen and systemic circulation, where it participates in cholesterol metabolism and lipogenesis [9]. After absorption into systemic circulation, propionic acid is transferred to the liver where it is used as a substrate for gluconeogenesis [20]. SCFAs also participate in the metabolism of glucose, lipids and cholesterol [21]. They are key components for maintaining gut-barrier integrity. The intestinal microbiota is capable of maintaining homeostasis, or it may contribute to disease susceptibility by changing the composition of GM metabolites that may affect host physiology [8]. Recent publications have indicated the relationship between dysbiosis, SCFA levels and genetic and immunologic factors that lead to the development of various conditions, mostly in adults, that reduce the patient’s quality of life. The conditions include diarrhea [22], obesity [23], irritable bowel syndrome (IBS) [24], inflammatory bowel diseases (IBDs), colon cancer [25], celiac disease [6] and non-alcoholic fatty liver disease (NAFLD), which may progress to non-alcoholic steatohepatitis (NASH) or even cirrhosis as a result of gut–liver axis malfunction and the accumulation of lipids in the liver [26,27,28]. Increased levels of trimethylamine-N-oxide (TMAO), a product of AA metabolism, may increase the risk of cardiovascular diseases by promoting atherosclerotic lesion development [29]. Lumen bacteria are also important in the development of depression because they can produce neurotransmitters such as serotonin and γ-aminobutyric acid (GABA) that are crucial in neuronal signaling [30]. Numerous studies have shown a direct link between dysbiosis and increased gut permeability, which allows the translocation of harmful compounds such as lipopolysaccharides (LPS) and pathogens to enter the inner layer of the intestinal barrier and, finally, to enter the bloodstream via the portal vein. This may disrupt the functioning of the gut–liver axis. It is related to the changes in SCFA production by the intestinal microbiome as it modulates the production of secretory immunoglobulin A (sIgA), a non-inflammatory antibody responsible for the prevention of pathogen invasion [31]. Inflammation caused by dysbiosis and pathogens, and that persists for a long time, may induce an inflammatory response that promotes liver injury, fibrosis, cirrhosis and oncogenic transformation, contributing to the development of diseases such as non-alcoholic fatty liver disease (NAFLD), non-alcoholic steatohepatitis (NASH), hepatocellular carcinoma, or primary hypertension (PH) [32,33]. NAFLD is the most common liver disease worldwide, estimated to affect up to 46% of the American population [34]. Bacteria with pro-inflammatory characteristics such as *Proteobacteria*, *Firmicutes*, or *Escherichia coli* are predominantly present, while protective bacteria such as *Faecalibacterium prausnitzii* are reduced in NAFLD patients [35]. At the bacterial family level, *Enterobacteriaceae* were reported to have increased, while *Rikenellaceae* and *Ruminococcaceae* were reported to have decreased. At the level of bacterial genera, *Escherichia*, *Dorea* and *Peptoniphilus* were reported to have increased, and *Anaerosporobacter*, *Coprococcus*, *Eubacterium*, *Faecalibacterium* and *Prevotella* were reported to have decreased [36]. The prevalence of NAFLD is growing due to an increasing number of people with obesity and related metabolic disorders, caused by unhealthy lifestyle, lack of exercise and an excessive intake of empty calories [37]. This results in insulin resistance due to decreased tissue sensitivity, which may ultimately lead to the development of type 2 diabetes. According to the latest data from the World Health Organization (WHO) from the year 2022, every one out of eight people is obese worldwide, and the number of obese adults has increased more than twice since 1990. It was also emphasized that up to 75% of obese adults and 50% of obese children developed metabolic disorders (Figure 2). Gut bacteria also produce endotoxins that can damage the liver and, thus, induce the promotion of NAFLD. There are three endotoxin-producing strains, i.e., *Enterobacter cloacae* B29, *Escherichia coli* PY102 and *Klebsiella pneumoniae* A7, which overgrow in the gut of morbidly obese patients and which have been shown to induce NAFLD when mono-associated with germ-free mice on a high-fat diet (HFD) [38]. Glucagon-like peptide-1 receptor (GLP-1) is another key factor in the regulation of body weight. It is responsible for promoting insulin secretion, insulin sensitivity and β-cell mass, while inhibiting gastric emptying and appetite, and affecting lipid intake [39]. The activity of the GLP-1 receptor can be regulated by the gastrointestinal microbiota due to the modulation of incretin hormone glucagon-like peptide-1 levels. Several strains of *Enterococcus faecalis* produce metabolites which decrease GLP-1 levels [40]. Conversely, all GLP-1-positive strains in the human gut were identified as *Staphylococcus epidermidis* by 16S rRNA sequencing [39].

## 3. Methodology of GC/MS Analysis

The most frequently used methods for SCFA analysis include nuclear magnetic resonance (NMR), gas chromatography (GC) and liquid chromatography (LC), with different types of detectors coupled with a mass spectrometer [20]. NMR is a highly reproducible method for the analyzed compound, but it is less sensitive than GC or LC methods, which have lower limits of detection (LOD) and quantification (LOQ) [41]. This is the main reason why GC and LC are more widely available in laboratories than NMR. Liquid chromatography is characterized by great resolution when complex matrices are analyzed, but GC is still the most commonly used method for SCFA analysis due to its reliability and accuracy [20,41]. Other advantages of GC/MS are the relative ease with which analyzed compound separation and identification may be achieved, and its high metabolic coverage [42].

### 3.1. Evidence from GC/MS-Based Studies

In this article, we presented a review of the literature of the analytic methods of SCFAs from studies conducted in the period from January 2018 to March 2024 using GC/MS as the main method of SCFA analysis in stool samples obtained from humans, animals and cell cultures. The summarized data from these publications are included in Table 1. Data extracted from studies using GC/MS methods are shown to highlight the latest developments in the field of SCFA analysis by GC/MS.

### 3.2. Sample Storage before Analysis

Numerous authors of the scientific research included in this review recommended storing stool samples at −80 °C to stop the metabolic activity of gut microbiome in the collected sample, and also to prevent the degradation of the analytes in the sample due to the activity of bacteria at room temperature, mostly microbial fermentation, and also aerobic conditions outside the intestine [42]. These are the main reasons why any steps taken before sample preparation for analysis should be performed at low temperatures, preferably on dry ice. Most samples are stored as raw feces, but Hsu et al. and Wang et al. used lyophilized samples for analysis [43,44]. However, the best option is to process fresh samples [45]. This is not an option in numerous analyses, so most researchers use frozen samples—it is more practical on a day-to-day basis. Only several protocols recommended the storage of samples at −20 °C in the form of fecal water [43] or of the stool sample in case of the acidification procedure [46]. Most studies analyzed in this review required sample storage at minus 80 °C to prevent changes in the composition of stool metabolome and to produce more accurate results obtained during GC analysis.

### 3.3. Sample Weight

The main factor driving the sample weight used for GC/MS analysis was the amount of the biologic sample obtained from the patient and the number of procedures in which it was used. With regard to the size, it is hard to obtain large amounts of feces from mice or rats. This factor drives the development of new, more accurate and reliable methods of SCFA quantification. The amount of feces used for analysis ranges from 10 milligrams in patients with pancreatitis [47] to 1 g in other methods of SCFA quantification [12,16]. The most common weights used for analysis are 50 and 100 milligrams of feces for human samples [49,50,51], and 10 to 50 milligrams for murine samples [44,52]. These values are most often selected because an analytical method must ensure appropriate levels of sensitivity and reproducibility so that the results obtained are reliable and truthful. To prevent the misinterpretation of acquired spectra, the addition of extraction blanks to the analyzed set samples is recommended [42].

### 3.4. Sample Preparation

Feces are a complex and diverse material that contains various metabolites resulting from the metabolism of intestinal bacteria, human metabolism and undigested food residues. Therefore, before starting the GC/MS analysis, SCFAs must be isolated from the sample. The basic method of SCFA extraction involves sample acidification by hydrochloric acid, phosphoric acid, formic acid, or sulfuric acid. It was developed to improve extraction efficiency and peak shape during GC analysis [43]. However, a faster loss of column quality is the main disadvantage of this method, contributing to higher analysis costs. To avoid this problem and extend the column life, sample acidification may be used with liquid–liquid extraction (LLE), which is the most frequently chosen method for SCFA isolation in this review. It uses organic solvents to make water-organic, two-phase solutions, which lead to the separation of analyzed compounds, e.g., chloroform, isobutanol, or ether. It is important to note that, because the charge of different compounds may vary with pH, there may not be one ideal pH value to suit all classes of compounds we would like to analyze. Therefore, any pH adjustment should be considered with respect to the experimental aim [42]. Suspending a stool sample in an organic solvent and its homogenization ensures proper fragmentation and the effective extraction of metabolites. Numerous researchers have added another step in sample preparation by using derivatization to prepare SCFA samples for analysis. By implementing this procedure, they obtained higher-purity samples for analysis [20]. Another advantage of this method is related to the fact that we obtain volatile compounds that are stable at high temperatures, in which GC/MS analysis is usually carried out. Methods involving derivatization are capable of achieving a lower limit of detection (LOD) and limit of quantification (LOQ) than previously used methods without derivatization [21]. The ideal derivatization agent should react selectively with the specific functional group, without any by-products of the reaction formed. The detection and separation of analyte derivatives should not be interfered with by the residues of the derivatization agent [53]. The most common approach presents silylation as the best way of preparing compounds for analysis. It results in the replacement of the acidic hydrogen atom with an alkylsilyl group [4] to form trimethylsilyl derivatives (TMS) using N-Methyl-N-(trimethyl-silyl)trifluoroacetamide (MSTFA) or bis(trimethylsilyl)trifluoroacetamide (BSTFA). These derivatives are highly volatile, stable and characterized by lower polarity, so they are more suited for GC analysis. If this method is used, we must remember that small, highly volatile compounds may evaporate from the sample during its preparation. Therefore, to avoid this, another modification of this method was developed: esterification using chloroformates, e.g., isobutyl chloroformate. In addition to the previously mentioned methods of preparing SCFAs for GC analysis, the latest and increasingly popular method is solid-phase extraction (SPE), or its more advanced version: solid-phase microextraction (SPME) [41]. This technique is faster, more selective and more sensitive due to the smaller amount of impurities in the sample [20]. SPME uses fibers for extraction, and it does not require a solvent to extract volatile compounds such as SCFAs. Due to the use of fibers, which are expensive and fragile, this method is more expensive than LLE with silylation [43]. It also requires additional laboratory equipment and specialist knowledge to be performed effectively [20]. Recently, researchers have more often combined solid-phase microextraction with derivatization into one method called headspace solid-phase microextraction (HS-SPME) [54]. Due to the better matrix clean-up and the reduction of interfering compounds, this method is more selective and has better sensitivity [20,55]. Such factors may also prolong the lifetime of the chromatographic system, thus lowering long-term analysis costs.

### 3.5. Internal Standard

During GC/MS analysis, an internal standard (IS) is added to each analyzed sample to check whether any metabolites have been lost during the extraction of the analyte from the sample [56]. The internal standard allows the compensation for the effects of the electron spray ionization (ESI) matrix effects, correction and normalization of the obtained results [20,57]. The best group of IS for targeted analysis of SCFAs includes isotopically labeled standards. Their use improves the specificity and precision of quantitative analysis [18,52]. In this study, deuterated standards were used most often [41,43,48,51,58,59,60], followed by 13C-labelled standards [20,41,52,54]. Other standards used for SCFA analysis are 2-methylvaleric acid [44,61], 4-methylvaleric acid [46], butyric acid esters [62,63], pivalic acid [64] and ribitol [56].

### 3.6. GC Parameters

The selection of appropriate GC analysis parameters is crucial for the effective separation of substances contained in the analyzed sample. It is most important to choose the appropriate chromatographic column—its length, width, type of stationary phase and its thickness. These parameters determine the efficiency of the column and its effectiveness in separating compounds with specific physicochemical properties. The length of the column and the thickness of the stationary phase film determine the duration of the analysis, i.e., the longer they are, the longer the separation time is. The width of the obtained peaks also increases. Almost all authors of articles included in this review used 30 m-long chromatography columns. Only Hough et al. [65] and Rui Wang et al. [21] used longer columns, i.e., 60 and 50 m, respectively. Shorter columns for analysis were used by Hoving et al. (25 m) [52], Kim et al. (15 m) [64] and Rohde et al. (15 m) [66]. For SCFA analysis, capillary columns with a non-polar stationary phase (5%-phenyl)-methylpolysiloxane are intended for the analysis of semi-volatile compounds (HP-5 column or analog), and high-polarity columns are intended for the analysis of volatile fatty acids, whose stationary phases are nitroterephthalic-acid-modified polyethylene glycol (DB-FFAP column or analog) or polyethylene glycol (DB-WAX column or analog). These types of columns are ideal for the separation of free fatty acids that are found in stool samples, especially in the case of methods without a derivatization procedure [21]. Therefore, three such types of column were used in almost all methods mentioned in Table 1. Gray et al. used DB-FATWAX Ultra Inert Polyethylene Glycol (PEG), DB-WAX and CP-Wax 58 FFAP columns. He stated that the DB-FATWAX column was far superior to two previously mentioned columns—it demonstrated consistent peak responses, retention times, sufficient resolution and no peak tailing over four times longer than the other two under acidic conditions, which are preferred due to the promotion of solubility and SCFA recoveries at low pH (1476 vs. 361 injections) [67]. Rohde et al. reported that succinic acid was a much better acidification agent than phosphoric acid because the recoveries obtained from it ranged from 95 to 117%, while from phosphoric acid they ranged from 111 to 177% [66].

Operating parameters such as the oven program, and carrier gas and its flow rate through the column, are also very important. The temperature at which the column operates determines the rate of the elution of the analyzed compounds from the column according to their increasing boiling points. Too low a temperature will result in the broadening of the peaks, while temperatures that are too high may lead to the overlapping of the peaks of the analyzed compounds. In both cases, performing a reliable analysis of the obtained results may be difficult. Overall, the main focus of researchers’ attention was to ensure optimal conditions for the separation of a mixture of short-chain fatty acids, which involved the experimental search for the best temperature parameters for analysis. In the present study, the scientists took a different approach when it came to the oven temperature program. Some of them assumed a shorter analysis time, like Yunkyung Kim et al., who used a very high ramp to decrease the analysis time to just 4.63 min, using nitrogen as a carrier gas and flame ionization detection [63], while the remaining researchers using FID detection ran times between 13.5 [68] and 24 min [64]. The fastest analysis using helium as a carrier gas was developed by Niccolai et al. and lasted 8.16 min [48]. Conversely, the longest one was developed by Jain et al. and lasted 56.81 min [56]. Looking chronologically, we can notice a tendency showing that, over time and with the appearance of subsequent publications, sample analysis times have shortened, which is undoubtedly related to the improvement of older methods, as well as the development of new ones due to the advances in the development of analytical research equipment.

The carrier gas used in GC analysis should be chemically neutral and should not interact with the analyzed compounds. It should also be of sufficient purity to prevent interference with the stationary phase, which could lead to a change in its properties. The viscosity and flow rate of the carrier gas exert a direct impact on the duration of the chromatographic analysis. The viscosity of hydrogen is twice as low compared to helium, making analysis using hydrogen twice as fast at the same flow. When selecting the carrier gas flow rate, the type of detector and its sensitivity, as well as the optimization of the analysis duration, should be considered. The most commonly used carrier gases include hydrogen, nitrogen, argon and helium, with helium usually being used in SCFA analysis, varying from 4N (99.99% purity) [21,65] to 6N (99.9999%). Nitrogen is used less commonly. Most authors chose helium at a flow rate of 1 mL/min, but some chose higher volumes, ranging from 1.1 [56] to 20 mL/min [58]. In most cases, a higher flow was used for shortening the time of analysis and sometimes to compensate for a slow/complex oven temperature program, which is important, when a laboratory has to analyze a large number of samples over a short period. Only Łoniewiska et al. used hydrogen as a carrier gas at a flow rate of 14.4 mL/min.

### 3.7. Detectors

In the development of a new chromatographic method, it is important to select a detector that is sufficiently sensitive and adapted to our needs. It should be characterized by high baseline stability and a wide range of linearity of concentration measurements. For the analysis of short-chain fatty acids, a flame ionization detector (FID) and an electron ionization detector (EID) are most often used. FID requires burning the sample in a hydrogen flame. This allows for changes in the electric potential of the resulting ions to be recorded. EIDs register charge that has been carried out by the fragmented compound over a certain period.

### 3.8. Mass Spectrometry Analysis

In gas chromatography–mass spectrometry, different modes of operation are used during sample analysis. A full scan mode is the most common one, with a mass spectrometer scanning a wide range of mass-to-charge ratios (m/z) to detect all ions present in the analyzed sample. The main advantage of this detection mode is that it allows the detection of all compounds present in the sample, providing a complete mass spectrum. It is ideal for untargeted analysis because of the collection of the data of all ions. Conversely, that is why this method is characterized by lower sensitivity and specificity. The selected ion monitoring (SIM) mode is the preferred mode for the analysis of specific ions of interest. It allows other ions to be ignored during the analysis. Therefore, this mode is characterized by high sensitivity due to instrument focus on specific ions, allowing for a better detection of low abundance compounds, high specificity due to the reduction of interference from other ions and better quantification due to the analysis of only specific ions. The multiple reaction monitoring (MRM) mode allows for the monitoring of specific precursor–product ion transitions for targeted compounds. It is primarily used in tandem mass spectrometry (MS/MS). The main advantages of the MRM mode include ultra-high specificity and sensitivity, and enhanced selectivity. Monitoring pairs of precursor–product ions allows background noise to be reduced and it improves detection limits. This mode can only be used for the analysis of known targets, and requires method development, which can be time-consuming.

## 4. Our Own Experience Regarding GC/MS Metabolomics

**Figure 3:**
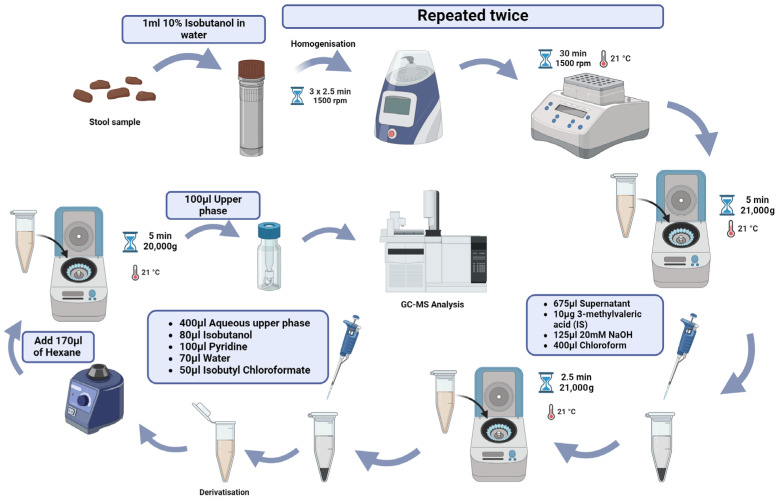
Sample preparation for GC/MS analysis in our protocol. Created with BioRender.com (accessed on 17 July 2024).

In our research, we utilized the GC/MS method to determine the concentrations of SCFAs and selected amino acids. Due to the nature of the study conducted by our team, the concentrations were measured in stool samples of various origins, including samples obtained from mice, healthy children, children with non-alcoholic fatty liver disease, obesity, or essential hypertension. Additionally, we examined samples from healthy adults, e-sports athletes, amateur and professional athletes, oncology patients with various types of cancer [77], patients with *Clostridium difficile* diarrheal cancer and inflammatory bowel disease. In our research, we used a method based on derivatization to determine SCFA levels in stool samples. The method used in our laboratory is shown in Figure 3. 

### 4.1. GC/MS Procedures

In brief, each collected stool sample was kept at −80 Celsius degrees before analysis. Next, the sample was weighed on dry ice (weighing 50–100 mg, depending on the origin: mouse or human) and was placed in a 2 mL tube containing ceramic beads designated for environmental sample analysis (Ohaus Corporation, Parsippany, NJ, USA). One milliliter of fresh 10% isobutanol solution was added to each sample. The samples were mechanically homogenized three times for 2.5 min each and then incubated at room temperature for 30 min. This procedure was performed twice. The homogenized samples were subsequently centrifuged at room temperature for 5 min at 21,000× *g*. A volume of 675 μL of the supernatant was collected and transferred to a new Eppendorf tube. To this, 10 μg of the internal standard (3-methyl valeric acid), 125 μL of 20 mM NaOH and 400 μL of chloroform were added. The sample was vortexed for 1 min and centrifuged for 2.5 min. A total of 400 μL of the upper aqueous phase was transferred to a new tube, followed by the addition of 100 μL of pyridine and 80 μL of isobutanol. The volume was adjusted to 650 μL with ultra-pure water. Calibration standards for SCFAs (formate, acetate, propionate, butyrate, isobutyrate and valerate) and amino acids (alanine, L-arginine, L-cystine, L-glutamic acid, L-leucine, L-lysine, L-serine, L-threonine, L-tyrosine, L-valine and L-histidine) were obtained from Sigma-Aldrich (St. Louis, MO). The derivatization of samples and standards was conducted with isobutyl chloroformate (50 μL per 650 μL sample or standard). The samples were vortexed in total for 1 min, followed by the addition of 170 μL of hexane, then vortexed again and centrifuged. The upper isobutyl-hexane phase was transferred to an autosampler vial for gas chromatographic analysis.

The analysis was performed with an Agilent 7000D Triple Quadrupole mass spectrometer coupled to a 7890 GC System with a G4513A autosampler (Agilent Technologies, Santa Clara, CA, USA) and a VF-5ms column (30 m, 0.25 mm, 0.50 μm). The injector, ion source, quadrupole and transfer line temperatures were set at 260 °C, 250 °C, 150 °C and 275 °C, respectively. Helium served as the carrier gas at a flow rate of 1 mL/min. The derivatized sample was injected into the VF-5ms column (Agilent Technologies, Santa Clara, CA, USA) with a split ratio of 50:1 and a solvent delay of 3 min. The oven temperature program was started at 40 °C for 5 min, increased to 275 °C at a rate of 10 °C/min and was maintained at this temperature for 10 min. The total run time was 38.5 min. MS data were collected in full scan mode from m/z 15 to 650 at 4.9 scans per second and analyzed using MassHunter software (version 10.1 build 10.1.733.0) (Agilent Technologies, Santa Clara, CA, USA). Finally, the obtained results were analyzed using standard biostatistics programs, such as GraphPad Prism.

### 4.2. Our Results Obtained during Studies Conducted Employing GC/MS

In this section, we present the findings from four publications [22,23,77,78] that explore SCFAs and amino acids levels which, alongside a variable microbiota, could potentially be recognized as biomarkers of diseases. It is worth noting that the discussed studies also address changes in the microbiota related to alpha and beta diversity. Identified bacteria and differentiated levels of SCFAs and AAs may serve as biomarkers of diseases, potentially contributing to diagnostics and influencing treatment outcomes. This detailed analysis offers valuable insights into the possible applications of these findings in clinical practice. 

In all GC/MS-based analyses of fecal samples discussed we identified seven SCFAs (acetic, butanoic, formic, hexanoic, isobutyric, pentanoic and propanoic acids) and nine amino acids (alanine, glycine, glutamic acid, isoleucine, leucine, methionine, phenylalanine, proline and valine).

The goal of the first study [78] was to investigate the impact of starch degradation products (SDexF) as prebiotics on obesity management in mice and overweight/obese children. We showed that SDexF reduced the relative fecal concentrations of pentanoic acid and all amino acids, while boosting the level of acetic acid in female mice on a normal diet and in male mice on a normal diet, respectively. In female mice on a Western diet, it led to an increase in propanoic acid and a reduction in alanine, valine, leucine, isoleucine and glutamic acid levels. Meanwhile, in male mice on a Western diet, there was an elevation in the levels of acetic acid, propanoic acid and butyric acid. In contrast to the significant effects of SDexF on weight gain, and on the gut microbiome and metabolome composition observed in the animal study, especially in female mice, SDexF did not influence weight loss or gut metagenomic and metabolomic profiles in children, with the only changes being noted in the abundance of specific taxa. However, a daily intake of vegetable and fruit mousses led to a notable reduction in relative amino acid levels by week 24 of the study, irrespective of SDexF treatment. This reduction was still partially evident at week 12, after the study concluded in both the prebiotic and control groups, though the clinical relevance of this finding remains uncertain. SDexF, known for its soluble fiber-like properties, is resistant to digestion by human enzymes due to its glycosidic bonds, which are not broken down by typical amylolytic enzymes. Consequently, it was anticipated that these compounds would pass through to the large intestine intact. Given SDexF’s known prebiotic effects, an increase in SCFA production was expected. However, while this effect was demonstrated in animal studies, it was not replicated in studies examining human feces.

In a subsequent study [23], we examined the composition and function of the gut microbiota, as well as the levels of SCFAs and AAs, in a group of 109 well-built Polish male sports players. The findings were compared with two reference groups: 25 endurance athletes and 36 healthy students of physical education. A six-week exercise training program in lean sedentary individuals resulted in an increase in fecal SCFA concentrations [79]. A literature review supported the view that exercise generally enhances the production of gut SCFAs. However, none of the SCFAs was distinct among the groups in our study. Instead, five SCFAs were associated with different enterotypes. Specifically, propanoic, isobutyric, pentanoic and hexanoic acids were linked to distinguishing between *Alistipes*- and *Bacteroides*-dominated enterotypes, while acetic and propanoic acids differentiated between *Prevotella*- and *Alistipes*-dominated enterotypes. Additionally, pentanoic acid was unique in distinguishing between *Prevotella*- and *Bacteroides*-dominated enterotypes. Increased bacterial metabolic activity in the distal colon may be influenced by a greater availability of amino acids. Unlike fecal SCFAs, all nine amino acids examined in this study showed differences between sports players and students. Furthermore, four amino acids varied between professional athletes and students, with methionine uniquely distinguishing sports players from both other groups. We also determined the correlation coefficients to assess the relationship between bacterial abundance and the levels of SCFAs and AAs. Specifically, *Bacteroides vulgaris*, *Barnesiella intestinihominis* and *Prevotella copri* were associated with at least five of the seven SCFAs studied. *Alistipes finegoldii* showed positive correlations with all nine AAs analyzed. In contrast, *Faecalibacterium prausnitzii* was negatively correlated with seven AAs.

The aim of the third study [22] was to compare the metagenomic and metabolomic profiles of patients with Clostridioides (*Clostridium*) *difficile*-associated diarrhea, cancer and inflammatory bowel disease (IBD). In this study, we used shotgun metagenomic sequencing and GC-MS to define the additive effect of *C. difficile* infection (CDI) on intestinal dysbiosis. In this study, we observed that the relative abundance of seven out of the nine measured fecal SCFAs distinguished at least two groups of diarrheal patients from the healthy control group. Specifically, formic acid and caproic acid were found in higher concentrations, while pentanoic acid was present in lower concentrations across all three diarrhea groups. Additionally, five amino acids showed differences between at least two patient groups and healthy controls. Among these, glycine and valine were more abundant, whereas methionine and glutamic acid were less abundant in each patient group.

To assess the relationship between the abundances of 56 species that distinguish all diarrheal patients from healthy controls, and 27 species that distinguish CDI patients from controls, we employed the Spearman correlation coefficient to analyze the levels of SCFAs and amino acids. Four specific species that separated diarrheal patients from those without diarrhea were identified: *Ruminococcus gnavus*, *E. coli* and *Klebsiella pneumoniae* showed a negative correlation with pentanoic acid levels. Both *E. coli* and *Klebsiella pneumoniae* were negatively correlated with glutamic acid but positively correlated with valine, while *Ruminococcus gnavus* and *E. coli* exhibited a positive correlation with phenylalanine. Most species that were under-represented in diarrheal patients showed a negative correlation with formic acid, isocaproic acid, glycine and valine, but a positive correlation with isobutyric acid, butanoic acid and pentanoic acid. In the case of CDI patients, eight species were under-represented compared to healthy controls. *Slackia isoflavoniconvertens*, *Blautia obeum*, *Ruminococcus torques*, *Dorea longicatena* and *CAG 139* showed negative correlations with isocaproic acid, while *Eubacterium ramulus*, *Blautia obeum* and *Dorea longicatena* were positively correlated with butanoic acid. *Bacteroides dorei*, *Blautia obeum* and *Ruminococcus torques* were correlated with methionine abundance. Among the 19 species that were over-represented in CDI patients, *Enterococcus faecalis*, *Lactobacillus rhamnosus* and *C. difficile* showed a positive correlation with isocaproic acid, whereas other metabolites were primarily correlated with individual bacterial species.

In the last of the studies discussed [77], we utilized shotgun sequencing along with GC/MS to perform metagenomic and metabolomic analyses. These techniques were employed to identify both common and distinct taxonomic configurations across patients with various cancers: 40 with colorectal cancer, 45 with stomach cancer, 71 with breast cancer, 34 with lung cancer, 50 with melanoma, 60 with lymphoid neoplasms and 40 with acute myeloid leukemia (AML). The findings were then compared to those from healthy controls (HC) who were matched for sex and age. In the present study, we discovered that fecal formic acid levels were significantly elevated across all seven case groups. Formate, an intermediate metabolite in one-carbon metabolism, facilitates metabolic interactions between mammals, their diet and the gut microbiome. It is produced by anaerobic fermentation from certain gut bacteria, and elevated levels in the gut lumen might indicate inflammation-related dysbiosis. In patients with breast cancer and colorectal cancer, there were increased levels of acetic, propanoic, isobutyric, butanoic and pentanoic acids, along with amino acids such as alanine, glycine and proline. In the group with lymphoid neoplasms, higher levels of isobutyric, pentanoic and hexanoic acids, as well as methionine and glutamic acid, were observed. However, in patients with lung cancer, stomach cancer, or melanoma, the concentrations of most fecal metabolites were similar to those of the control group. We recalculated the correlation coefficients between the bacteria differentiating the studied patient groups and HC, and the levels of bacterial metabolites. Most of the species that were over-represented in our case samples showed a positive correlation with fecal levels of valine, phenyloalanine and glycine, and a negative correlation with hexanoic acid levels. In contrast, bacterial species that were more abundant in control samples exhibited the opposite correlations. Additionally, a sub-group of seven species of *Faecalibacterium* correlated negatively with most AAs and formic acid, but positively with acetic, propanoic and butanoic acid levels.

As evidenced by the results of our own research, determining the correlations between bacteria and their produced metabolites appears to be a more effective tool for identifying biomarkers of various diseases. Integrating such studies into routine patient therapy and monitoring could potentially enhance diagnostic accuracy and treatment outcomes. By using these insights, healthcare providers can better tailor treatment plans and improve patient outcomes through a deeper understanding of microbial interactions. Further research in this area could lead to significant advancements in personalized medicine, emphasizing the importance of the gut microbiome in overall health.

## 5. Conclusions

Fecal metabolite analysis is gaining prominence in metabolomics due to its comprehensive metabolic insights and the accessibility of fecal samples. GC-MS is a highly effective technique. This method allows for accurate quantitative analysis, determining the exact concentrations of components within a sample, and provides a qualitative identification of compounds, which is essential across various scientific disciplines. The rapid separation process in gas chromatography facilitates quick results. Additionally, the automation of many GC-MS systems enhances efficiency and minimizes errors from manual sample handling. MS excels at distinguishing and analyzing closely related chemical compounds—a task that may be difficult for other analytical methods. GC is particularly adept at separating and analyzing volatile and semi-volatile substances, making it invaluable in numerous industrial and research contexts. Our review covers fecal metabolomics in human studies, highlighting common metabolic patterns associated with different diets and health conditions. We examined the available literature on the methods for analyzing SCFAs in stool samples obtained from humans, animals and cell cultures. The key parameters of the studies we reviewed included sample storage before analysis, sample weight, sample preparation methods, the use of internal standards, GC parameters, types of detector used and MS parameters. 

GC-MS has long been a preferred method for analyzing volatile compounds in biological samples, including feces. In thermal desorption GC-MS, volatiles from the headspace of heated feces are absorbed onto a chosen medium or trapped directly, then released by heating, and injected into the GC column for MS detection. A common absorbent used is the polymer-coated fiber of an SPME system, with extraction effectiveness depending on the fiber type and extraction duration. For non-volatile compounds, chemical derivatization is essential in GC-MS studies to convert non-volatile forms into volatile ones suitable for GC analysis. Before derivatization, an extraction process is necessary to isolate the desired compounds from the biospecimen. Developing a robust and standardized fecal metabolomics methodology, including precise quantitation and identification of biomarkers, is crucial for advancing fecal metabolomics into wider clinical applications.

## Figures and Tables

**Table 1:** Review of methodological literature from 2018 to 2024.

	Publication	Condition/ Purpose	Compounds/ Material	Extraction	Column	Gas/Flow	Oven T [°C]	Derivatization Agent	Run Time [min]	MS Mode	Inlet [°C]	Transfer Line [°C]	Ion Source [°C]
1	Chaozheng Zhang et al. [41] 2018	Method development	SCFA/feces GC/HS	LLE	HP-innowax capillary column with polyethylene glycol as stationary phase (30 m × 0.32 mm × 0.50 µm)	Nitrogen 33 cm/s	150–190 °C—5 °C/min 190–210 °C—20 °C/min 210 °C—1 min	-	10	EI+	200 °C	-	-
2	Jessica Fiori et al. [42] 2018	Microbiota metabolic profile	SCFA/feces	HS-SPME	Phenomenex ZB-WAX (30 m × 0.25 mm × 0.15 µm)	Helium 1 mL/min	40 °C—5 min 40–220 °C—10 °C/min 220 °C—5 min	-	28	EI+	250 °C	250 °C	200 °C
3	Liqing He et al. [43] 2018	Method development	SCFA/feces	LLE	DB-225ms column (30 m × 0.25 mm × 0.25 μm) DB-5ms (30 m × 0.25 mm × 0.25 μm)	Helium 1.5 mL/min or 1.0 mL/min (both columns together)	80 °C—0.5 min 80–158 °C—10 °C/min 158–160 °C—3 °C/min 160–220 °C—20 °C/min 220 °C—8 min	PFBBr	20.63	EI+	220 °C	220 °C	220 °C
4	Lisa R. Hoving et al. [44] 2018	Method development	SCFA/feces	LLE	VF-5 ms column (25 m × 0.25 mm × 0.25 μm)	Helium 1.2 mL/min	40 °C—1 min 40–60 °C—40 °C/min 60 °C—3 min 60–210 °C—25 °C/min 210–315 °C—40 °C/min 315 °C—3 min	-	15.13	-	280 °C	280 °C	280 °C
5	Rachael Hough et al. [45] 2018	Method development	VOCs/feces	HS-SPME	Zebron ZB-624 GC (60 m × 0.25 mm × 1.4 µm)	Helium 1 mL/min	40 °C—1 min 40–220 °C—5 °C/min 220 °C—4 min	-	41	EI+	-	-	-
6	Takeshi Furuhashi et al. [46] 2018	Method development	SCFA/feces	LLE	HP-5 ms capillary column (30 m × 250 μm × 0.25 μm)	-	50 °C—5 min 50–150 °C—5 °C/min 150–330 °C—40 °C/min 330 °C—1 min	chloroformate (propyl/isobutyl/1-butyl-)	30.5	EI+	-	250 °C	250 °C
7	Xue Han et al. [47] 2018	Mice	SCFA/feces	LLE	HP-FFAP (30 m × 0.25 mm × 0.25 μm)	Helium	90 °C 90–150 °C—12 °C/min 150–220 °C—20 °C/min 220 °C—4.5 min	-	13	TIC EI+	175 °C	220 °C	230 °C
8	Abhishek Jain et al. [48] 2019	Human metabolism	SCFA/feces	LLE	HP-5MS capillary 54 column (30 m × 0.250 mm × 0.25 μm)	Helium 1.1 mL/min	75 °C—4 min 75–280 °C—4 °C/min 280 °C—1.56 min	MSTFA TMCS	56.81	EI+	250 °C	-	230 °C
9	Caroline Douny et al. 2019 [49]	Gastrointestinal model	SCFA/feces	SPME	Supelcowax-10 column (30 m × 0.25 mm × 0.2 μm)	Helium	50 °C—5.5 min 50–175 °C—75 °C/min 175 °C—2 min 175–200 °C—10 °C/min 200 °C—3 min	-	15.1	EI+	250 °C	230 °C	220 °C
10	Elena Niccolai et al. [50] 2019	Gut diseases	SCFA/feces	LLE	Supelco Nukol column (30 m × 0.25 mm × 0.25 µm)	Helium 1 mL/min	40 °C—1 min 40–150 °C—30 °C/min 150–220 °C—20 °C/min	-	8.16	-	250 °C	280 °C	-
11	Hayoung Kim et al. [51] 2019	Method development	SCFA/microbes	LLE	Nukol™ capillary GC column (15 m × 0.53 mm × 0.5 μm)	Nitrogen 40 mL/min	80 °C—2 min 80–190 °C—5 °C/min	TBDMS	24	FID	190 °C	-	-
12	M.A. López-Bascón et al. [52] 2019	Pig feces	SCFA/feces	LLE	DB-5MS-UI column (30 m × 0.25 mm × 0.25 µm)	Helium 1 mL/min	60 °C—1 min 60–300 °C—10 °C/min 300 °C—2 min	BSTFA TMCS	27	EI+	250 °C	280 °C	300 °C
13	Shuming Zhang et al. [53] 2019	Method development	SCFA/feces	LLE	HB-5 ms capillary column (30 m × 0.25 mm × 0.25 µm)	Helium 1 mL/min	40 °C—2 min 40–150 °C—15 °C/min 150 °C—1 min 150–300 °C—30 °C/min 300 °C—5 min	BSTFA	20.20	EI+	250 °C	280 °C	230 °C
14	Ya-Lin Hsu et al. [54] 2019	Method development	SCFA/feces	LLE	VF-WAXms capillary column (30 m × 0.25 mm × 0.25 μm)	Helium 1 mL/min	70 °C—1 min 70–170 °C—10 °C/min 170–240 °C—20 °C/min 240 °C—2 min	-	15.8	EI+	250 °C	250 °C	240 °C
15	Zhixing He et al. [55] 2019	Ankylosing spondylitis	SCFA/feces	LLE	HP-5 ms capillary column (30 m × 0.25 mm × 0.25 μm)	Helium 1.2 mL/min	80 °C—2 min 80–330 °C—10 °C/min 330 °C—6 min	methoxyamine hydrochloride	33	-	280 °C	-	230 °C
16	Chaozheng Zhang et al. [56] 2020	Mice	SCFA/feces	LLE	DB-FFAP capillary column with polyethylene glycol modified by terephthalic acid as stationary phase (30 m × 0.32 mm × 0.5 µm)	Nitrogen 2 mL/min	90 °C—5 min 90–200 °C—20 °C/min 200 °C—3 min	-	13.5	FID	250 °C	-	-
17	Huan Wang et al. [57] 2020	Type 2 Diabetic Rats	SCFA/feces	LLE	DB-FFAP (30 m × 0.25 mm × 0.25 μm)	Helium 1 mL/min	100 °C—3 min 100–150 °C—5 °C/min 150–200 °C—20 °C/min 200 °C—5 min	-	22.5	EI−	250 °C	250 °C	230 °C
18	Menghan Li et al. [18] 2020	Method development	SCFA/feces	LLE	DB-5 MS UI capillary column (30 m × 0.25 mm × 0.25 μm)	Helium 1.2 mL/min	70 °C—3 min 70–200 °C—10 °C/min 200–295 °C—35 °C/min 295 °C—7 min	BCF	25.5	EI−	250 °C	280 °C	230 °C
19	Rui Wang et al. [21] 2020	Digestive diseases	SCFA/feces	LLE	DB-FFAP (50 m × 0.32 mm × 0.5 μm)	Helium 1 mL/min	70 °C 70–180 °C—10 °C/min 180–200 °C—5 °C/min	-	22	EI+	250 °C	230 °C	230 °C
20	Sofa el Manouni el Hassani et al. [58] 2020	Method development	SCFA/feces	LLE	Restek RTX-1 capillary column (30 m × 0.32 mm × 4 μm)	-	10 °C—8.22 min 10–280 °C—25 °C/min 280 °C—2 min	-	21.02	FID	-	-	-
21	Zhenyi Tian et al. [59] 2020	Diarrhea-predominant irritable bowel syndrome	SCFA/feces	HS-SPME	Supelcowax 10 capillary column (30 m × 0.25 mm × 0.25 μm)	Helium 1.3 mL/min	100–120 °C—5 °C/min 120–150 °C—2 °C/min 150–240 °C—30 °C/min 240 °C—1 min	-	23	EI+	250 °C	280 °C	200 °C
22	B. Loye Eberhart II et al. [60] 2021	Method development	SCFA/feces	LLE	DB-FFAP (30 m × 0.53 mm × 0.50 μm)	Helium 3 mL/min	40 °C—1 min 40–250 °C—20 °C/min 250 °C—10 min	-	21.5	-	240 °C	-	-
23	Daiki Watanabe et al. [61] 2021	Tumorigenic bacteria	SCFA/feces	LLE	Shimadzu BPX5 column (30 m × 0.25 mm × 0.25 μm)	Helium 1.2 mL/min	-	MTBSTFA	-	-	230 °C	260 °C	-
24	Daniel van der Lelie et al. [62] 2021	Immune-mediated colitis	SCFA/feces	LLE	DB-5ms column (30 m × 0.25 mm × 0.25 μm)	Helium 1.0 mL/min	50 °C—2 min 50–70 °C—10 °C/min 70–85 °C—3 °C/min 85–110 °C—5 °C/min 110–290 °C—30 °C/min 290 °C—8 min	PCF	28	-	-	-	-
25	Haiwei Gu et al. [63] 2021	Method development	SCFA/feces	LLE	HP-5 ms capillary column (30 m × 0.25 mm × 0.25 μm)	Helium 20 mL/min	60 °C—1 min 60–325 °C—10 °C/min 325 °C—10 min	MTBSTFA	37.5	EI+	250 °C	290 °C	230 °C
26	Justin Gray et al. [64] 2021	Method development	SCFA/feces	LLE	Hydroguard Water-Resistant Guard Column (5 m × 0.25 mm) DB-FATWAX Ultra Inert PEG Column (30 m × 0.25 mm × 0.25 μm) + Vu2 Column Union	Helium 1.5 mL/min	80 °C—2.5 min 80–230 °C—15 °C/min 230–245 °C—30 °C/min 245 °C—2 min	-	15	EI+	250 °C	-	-
27	Miftakh Nur Rahman et al. [65] 2021	Central obesity	SCFA/serum	LLE	Nukol-fused silica capillary column (30 m × 0.25 mm × 0.25 µm)	Helium 2.29 mL/min	60–180 °C—10 °C/min 180 °C—12 min	-	25	-	-	200 °C	200 °C
28	Julia K. Rohde et al. [66] 2022	Method development	SCFA/feces	LLE	Nukol-fused silica capillary column (15 m × 0.32 mm × 0.25 µm)	Helium 2.5 mL/min-6.2 min, 5 mL/min—5.1 min	55 °C—1 min 55–105 °C—8 °C/min 105 °C—2 min 105–190 °C—30 °C/min 190 °C—1 min	-	13	EI+	200 °C	200 °C	250 °C
29	Kyeong-Seog Kim et al. [67] 2022	Method development	SCFA/feces	LLE	DB-FFAP column (30 m × 0.25 mm × 0.25 µm)	Helium 1 mL/min	40 °C—2 min 40–95 °C—40 °C/min 95 °C—1 min 95–140 °C—5 °C/min 140–200 °C—40 °C/min	-	15	EI+	-	280 °C	230 °C
30	Lin Li et al. [68] 2022	Idiopathic Short Stature	SCFA/feces	LLE	HP-INNOWAX capillary GC column (30 m × 0.25 mm × 0.25 µm)	Helium 1.0 mL/min	90–120 °C—10 °C/min 120–150 °C—5 °C/min 150–250 °C—25 °C/min 250 °C—2 min	-	15	-	250 °C	-	230 °C
31	Victoria Ramos-Garcia et al. [69] 2022	Newborns and lactating mothers	SCFA/feces	LLE	HP-5 ms capillary column (30 m × 0.25 mm × 0.25 μm)	Helium 1 mL/min	50 °C—2 min 50–70 °C—10 °C/min 70–85 °C—3 °C/min 85–110 °C—5 °C/min 110–290 °C—30 °C/min 290 °C—8 min	PCF	28	EI-	260 °C	290 °C	230 °C
32	Łoniewska et al. [15] 2023	Newborns	SCFA/feces	LLE	DB-FFAP column (30 m × 0.53 mm × 0.5 μm)	Hydrogen 14.4 mL/min	100 °C—0.5 min 100–180 °C—8 °C/min 180 °C—1 min 180–200 °C—20 °C/min 200 °C—5 min	-	17.5 min				
33	Isabela Solar et al. [70] 2023	Obesity	SCFA/feces	LLE	Stabilwax capillary column (30 m × 0.25 mm × 0.25 μm)	Helium 1.0 mL/min	-	-	-	EI+	250 °C	-	200 °C
34	Jia Jia Xu et al. [71] 2023	Pancreatitis	SCFA/feces	LLE	Rxi-5MS column (30 m × 0.25 mm × 0.25 μm)	Helium 1.0 mL/min	45 °C—1 min 45–260 °C—20 °C/min 260–320 °C—40 °C/min 320 °C—2 min		15.25	EI+	270 °C	270 °C	220 °C
35	Sunhee Kang et al. [72] 2023	Mice	SCFA/feces	SPME	DB WAXetr capillary column (30 m × 0.25 mm × 0.25 µm)	Helium 1 mL/min	80 °C—2 min 80–100 °C—10 °C/min 100–130 °C—5 °C/min 130–160 °C—10 °C/min 160–220 °C—20 °C/min 220 °C—2 min	-	16	EI	-	240 °C	230 °C
36	YiZhong Wang et al. [73] 2023	Glycogen storage disease (GSD)	SCFA/feces	LLE	HP FFAP capillary column (30 m × 0.25 mm × 0.25 µm)	Helium 1.0 mL/min	40 °C—2 min 40–150 °C—15 °C/min 150 °C—1 min 150–300 °C—30 °C/min 300 °C—5 min	-	20.33	EI+	260 °C	280 °C	230 °C
37	Yunkyung Kim et al. [74] 2023	Fibromyalgia	SCFA/feces	LLE	HP-innowax capillary GC column (30 m × 0.32 mm × 0.25 µm)	Nitrogen	60–170 °C—30 °C/min 170–180 °C—40 °C/min 180 °C—0.75 min	-	4.63	FID	90 °C	100 °C	250 °C
38	Mya Thandar et al. [75] 2024	Colorectal fibrosis	SCFA/feces	LLE	DB-5MS fused silica capillary column (30 m × 0.25 mm × 0.25 μm)	Helium 1.0 mL/min	60 °C—0.5 min 60—305 °C 305 °C—5 min	BSTFA	-	EI+	260 °C	-	230 °C
39	Tianqu Xie et al. [76] 2024	Prenatal depression	SCFA/feces	LLE	HP FFAP capillary column (30 m × 0.25 mm × 0.25 μm)	Helium 1.0 mL/min	80–120 °C—40 °C/min 120–230 °C—10 °C/min 230 °C—3 min	-	15	EI	260 °C	230 °C	230 °C

BCF—Benzyl chloroformate; BSTFA—N,O-Bis(trimethylsilyl)trifluoroacetamide; MSTFA—N-methyl-N-(trimethylsilyl)-trifluoroacetamide; TMCS—trimethylchlorosilane; MTBSTFA—N-tert-butyldimethylsilyl-N-methyltrifluoroacetamide; PCF—propyl-chloroformate; TBDMS—tert-Butyldimethylsilyl chloride; PFBBr—pentafluorobenzyl bromide.

## Data Availability

No new data were created or analyzed in this study.

## References

[1] Krassowski M., Das V., Sahu S.K., Misra B.B. (2020). State of the Field in Multi-Omics Research: From Computational Needs to Data Mining and Sharing. Front. Genet..

[2] Dong X., Liu C., Dozmorov M. (2021). Review of Multi-Omics Data Resources and Integrative Analysis for Human Brain Disorders. Brief. Funct. Genom..

[3] Santiago-Rodriguez T.M., Hollister E.B. (2021). Multi ‘omic Data Integration: A Review of Concepts, Considerations, and Approaches. Semin. Perinatol..

[4] Liu J., Tan Y., Cheng H., Zhang D., Feng W., Peng C. (2022). Functions of Gut Microbiota Metabolites, Current Status and Future Perspectives. Aging Dis..

[5] Magnúsdóttir S., Ravcheev D., de Crécy-Lagard V., Thiele I. (2015). Systematic Genome Assessment of B-Vitamin Biosynthesis Suggests Co-Operation among Gut Microbes. Front. Genet..

[6] Gasaly N., de Vos P., Hermoso M.A. (2021). Impact of Bacterial Metabolites on Gut Barrier Function and Host Immunity: A Focus on Bacterial Metabolism and Its Relevance for Intestinal Inflammation. Front. Immunol..

[7] Zhgun E.S., Ilyina E.N. (2020). Fecal Metabolites As Non-Invasive Biomarkers of Gut Diseases. Acta Nat..

[8] Fiori J., Turroni S., Candela M., Gotti R. (2020). Assessment of Gut Microbiota Fecal Metabolites by Chromatographic Targeted Approaches. J. Pharm. Biomed. Anal..

[9] Tsukuda N., Yahagi K., Hara T., Watanabe Y., Matsumoto H., Mori H., Higashi K., Tsuji H., Matsumoto S., Kurokawa K. (2021). Key Bacterial Taxa and Metabolic Pathways Affecting Gut Short-Chain Fatty Acid Profiles in Early Life. ISME J..

[10] Bäckhed F., Roswall J., Peng Y., Feng Q., Jia H., Kovatcheva-Datchary P., Li Y., Xia Y., Xie H., Zhong H. (2015). Dynamics and Stabilization of the Human Gut Microbiome during the First Year of Life. Cell Host Microbe.

[11] Dogra S., Sakwinska O., Soh S.-E., Ngom-Bru C., Brück W.M., Berger B., Brüssow H., Lee Y.S., Yap F., Chong Y.-S. (2015). Dynamics of Infant Gut Microbiota Are Influenced by Delivery Mode and Gestational Duration and Are Associated with Subsequent Adiposity. mBio.

[12] Stokholm J., Blaser M.J., Thorsen J., Rasmussen M.A., Waage J., Vinding R.K., Schoos A.-M.M., Kunøe A., Fink N.R., Chawes B.L. (2018). Maturation of the Gut Microbiome and Risk of Asthma in Childhood. Nat. Commun..

[13] Nilsen M., Nygaard U.C., Brodin P., Carlsen K.C.L., Fredheim C., Haugen G., Hedlin G., Jonassen C.M., Jonsmoen U.L.A., Lakshmikanth T. (2024). Gut Bacteria at 6 Months of Age Are Associated with Immune Cell Status in 1-Year-Old Children. Scand. J. Immunol..

[14] Vatanen T., Franzosa E.A., Schwager R., Tripathi S., Arthur T.D., Vehik K., Lernmark Å., Hagopian W.A., Rewers M.J., She J.-X. (2018). The Human Gut Microbiome in Early-Onset Type 1 Diabetes from the TEDDY Study. Nature.

[15] Łoniewska B., Fraszczyk-Tousty M., Tousty P., Skonieczna-Żydecka K., Maciejewska-Markiewicz D., Łoniewski I. (2023). Analysis of Fecal Short-Chain Fatty Acids (SCFAs) in Healthy Children during the First Two Years of Life: An Observational Prospective Cohort Study. Nutrients.

[16] Kemp K.M., Orihuela C.A., Morrow C.D., Judd S.E., Evans R.R., Mrug S. (2024). Associations between Dietary Habits, Socio-Demographics and Gut Microbial Composition in Adolescents. Br. J. Nutr..

[17] Nicholson J.K., Holmes E., Kinross J., Burcelin R., Gibson G., Jia W., Pettersson S. (2012). Host-Gut Microbiota Metabolic Interactions. Science.

[18] Li X., Shimizu Y., Kimura I. (2017). Gut Microbial Metabolite Short-Chain Fatty Acids and Obesity. Biosci. Microbiota Food Health.

[19] Whisner C.M., Castillo L.F. (2018). Prebiotics, Bone and Mineral Metabolism. Calcif. Tissue Int..

[20] Primec M., Mičetić-Turk D., Langerholc T. (2017). Analysis of Short-Chain Fatty Acids in Human Feces: A Scoping Review. Anal. Biochem..

[21] Wang R., Fan C., Fan X., Zhao Y., Wang Y., Li P., Tang T., Yao H., Chen S., Chen D. (2020). A Fast and Accurate Way to Determine Short Chain Fatty Acids in Human Serum by GC–MS and Their Distribution in Children with Digestive Diseases. Chromatographia.

[22] Kulecka M., Zeber-Lubecka N., Bałabas A., Czarnowski P., Bagińska K., Głowienka M., Kluska A., Piątkowska M., Dąbrowska M., Waker E. (2023). Diarrheal-Associated Gut Dysbiosis in Cancer and Inflammatory Bowel Disease Patients Is Exacerbated by Clostridioides Difficile Infection. Front. Cell Infect. Microbiol..

[23] Kulecka M., Fraczek B., Balabas A., Czarnowski P., Zeber-Lubecka N., Zapala B., Baginska K., Glowienka M., Szot M., Skorko M. (2023). Characteristics of the Gut Microbiome in Esports Players Compared with Those in Physical Education Students and Professional Athletes. Front. Nutr..

[24] Sun Q., Jia Q., Song L., Duan L. (2019). Alterations in Fecal Short-Chain Fatty Acids in Patients with Irritable Bowel Syndrome: A Systematic Review and Meta-Analysis. Medicine.

[25] Carretta M.D., Quiroga J., López R., Hidalgo M.A., Burgos R.A. (2021). Participation of Short-Chain Fatty Acids and Their Receptors in Gut Inflammation and Colon Cancer. Front. Physiol..

[26] Han H., Jiang Y., Wang M., Melaku M., Liu L., Zhao Y., Everaert N., Yi B., Zhang H. (2023). Intestinal Dysbiosis in Nonalcoholic Fatty Liver Disease (NAFLD): Focusing on the Gut–Liver Axis. Crit. Rev. Food Sci. Nutr..

[27] Chen J., Vitetta L. (2020). Gut Microbiota Metabolites in NAFLD Pathogenesis and Therapeutic Implications. Int. J. Mol. Sci..

[28] Kobayashi T., Iwaki M., Nakajima A., Nogami A., Yoneda M. (2022). Current Research on the Pathogenesis of NAFLD/NASH and the Gut–Liver Axis: Gut Microbiota, Dysbiosis, and Leaky-Gut Syndrome. Int. J. Mol. Sci..

[29] Ussher J.R., Lopaschuk G.D., Arduini A. (2013). Gut Microbiota Metabolism of L-Carnitine and Cardiovascular Risk. Atherosclerosis.

[30] McCarville J.L., Chen G.Y., Cuevas V.D., Troha K., Ayres J.S. (2020). Microbiota Metabolites in Health and Disease. Annu. Rev. Immunol..

[31] Takeuchi T., Ohno H. (2021). Reciprocal Regulation of IgA and the Gut Microbiota: A Key Mutualism in the Intestine. Int. Immunol..

[32] Chopyk D.M., Grakoui A. (2020). Contribution of the Intestinal Microbiome and Gut Barrier to Hepatic Disorders. Gastroenterology.

[33] Masarone M., Troisi J., Aglitti A., Torre P., Colucci A., Dallio M., Federico A., Balsano C., Persico M. (2021). Untargeted Metabolomics as a Diagnostic Tool in NAFLD: Discrimination of Steatosis, Steatohepatitis and Cirrhosis. Metabolomics.

[34] Lechner S., Yee M., Limketkai B.N., Pham E.A. (2020). Fecal Microbiota Transplantation for Chronic Liver Diseases: Current Understanding and Future Direction. Dig. Dis. Sci..

[35] Grabherr F., Grander C., Effenberger M., Adolph T.E., Tilg H. (2019). Gut Dysfunction and Non-Alcoholic Fatty Liver Disease. Front. Endocrinol..

[36] Aron-Wisnewsky J., Vigliotti C., Witjes J., Le P., Holleboom A.G., Verheij J., Nieuwdorp M., Clément K. (2020). Gut Microbiota and Human NAFLD: Disentangling Microbial Signatures from Metabolic Disorders. Nat. Rev. Gastroenterol. Hepatol..

[37] Iruzubieta P., Medina J.M., Fernández-López R., Crespo J., De La Cruz F. (2020). A Role for Gut Microbiome Fermentative Pathways in Fatty Liver Disease Progression. JCM.

[38] Fei N., Bruneau A., Zhang X., Wang R., Wang J., Rabot S., Gérard P., Zhao L. (2020). Endotoxin Producers Overgrowing in Human Gut Microbiota as the Causative Agents for Nonalcoholic Fatty Liver Disease. mBio.

[39] Tomaro-Duchesneau C., LeValley S.L., Roeth D., Sun L., Horrigan F.T., Kalkum M., Hyser J.M., Britton R.A. (2020). Discovery of a Bacterial Peptide as a Modulator of GLP-1 and Metabolic Disease. Sci. Rep..

[40] LeValley S.L., Tomaro-Duchesneau C., Britton R.A. (2020). Degradation of the Incretin Hormone Glucagon-Like Peptide-1 (GLP-1) by Enterococcus Faecalis Metalloprotease GelE. mSphere.

[41] Zhang C., Tang P., Xu H., Weng Y., Tang Q., Zhao H. (2018). Analysis of Short-Chain Fatty Acids in Fecal Samples by Headspace-Gas Chromatography. Chromatographia.

[42] Fiori J., Turroni S., Candela M., Brigidi P., Gotti R. (2018). Simultaneous HS-SPME GC-MS Determination of Short Chain Fatty Acids, Trimethylamine and Trimethylamine N-Oxide for Gut Microbiota Metabolic Profile. Talanta.

[43] He L., Prodhan M.A.I., Yuan F., Yin X., Lorkiewicz P.K., Wei X., Feng W., McClain C., Zhang X. (2018). Simultaneous Quantification of Straight-Chain and Branched-Chain Short Chain Fatty Acids by Gas Chromatography Mass Spectrometry. J. Chromatogr. B.

[44] Hoving L.R., Heijink M., Van Harmelen V., Van Dijk K.W., Giera M., Giera M. (2018). GC-MS Analysis of Short-Chain Fatty Acids in Feces, Cecum Content, and Blood Samples. Clinical Metabolomics.

[45] Hough R., Archer D., Probert C. (2018). A Comparison of Sample Preparation Methods for Extracting Volatile Organic Compounds (VOCs) from Equine Faeces Using HS-SPME. Metabolomics.

[46] Furuhashi T., Sugitate K., Nakai T., Jikumaru Y., Ishihara G. (2018). Rapid Profiling Method for Mammalian Feces Short Chain Fatty Acids by GC-MS. Anal. Biochem..

[47] Han X., Guo J., You Y., Yin M., Ren C., Zhan J., Huang W. (2018). A Fast and Accurate Way to Determine Short Chain Fatty Acids in Mouse Feces Based on GC–MS. J. Chromatogr. B.

[48] Jain A., Li X.H., Chen W.N. (2019). An Untargeted Fecal and Urine Metabolomics Analysis of the Interplay between the Gut Microbiome, Diet and Human Metabolism in Indian and Chinese Adults. Sci. Rep..

[49] Douny C., Dufourny S., Brose F., Verachtert P., Rondia P., Lebrun S., Marzorati M., Everaert N., Delcenserie V., Scippo M.-L. (2019). Development of an Analytical Method to Detect Short-Chain Fatty Acids by SPME-GC–MS in Samples Coming from an in Vitro Gastrointestinal Model. J. Chromatogr. B.

[50] Niccolai E., Baldi S., Ricci F., Russo E., Nannini G., Menicatti M., Poli G., Taddei A., Bartolucci G., Calabrò A.S. (2019). Evaluation and Comparison of Short Chain Fatty Acids Composition in Gut Diseases. WJG.

[51] Kim H., Kwon J., Choi S.Y., Ahn Y.G. (2019). Method Development for the Quantitative Determination of Short Chain Fatty Acids in Microbial Samples by Solid Phase Extraction and Gas Chromatography with Flame Ionization Detection. J. Anal. Sci. Technol..

[52] López-Bascón M.A., Calderón-Santiago M., Argüello H., Morera L., Garrido J.J., Priego-Capote F. (2019). Comprehensive Analysis of Pig Feces Metabolome by Chromatographic Techniques Coupled to Mass Spectrometry in High Resolution Mode: Influence of Sample Preparation on the Identification Coverage. Talanta.

[53] Zhang S., Wang H., Zhu M.-J. (2019). A Sensitive GC/MS Detection Method for Analyzing Microbial Metabolites Short Chain Fatty Acids in Fecal and Serum Samples. Talanta.

[54] Hsu Y.-L., Chen C.-C., Lin Y.-T., Wu W.-K., Chang L.-C., Lai C.-H., Wu M.-S., Kuo C.-H. (2019). Evaluation and Optimization of Sample Handling Methods for Quantification of Short-Chain Fatty Acids in Human Fecal Samples by GC–MS. J. Proteome Res..

[55] He Z., Wang M., Li H., Wen C. (2019). GC-MS-Based Fecal Metabolomics Reveals Gender-Attributed Fecal Signatures in Ankylosing Spondylitis. Sci. Rep..

[56] Zhang C., Liu A., Zhang T., Li Y., Zhao H. (2020). Gas Chromatography Detection Protocol of Short-Chain Fatty Acids in Mice Feces. Bio-Protocol.

[57] Wang H., Liu Y., Shao J., Luo Y., Cai W., Chen L. (2020). Rapid and Accurate Simultaneous Determination of Seven Short-Chain Fatty Acids in Feces by Gas Chromatography—Mass Spectrometry (GC-MS): Application in Type 2 Diabetic Rats and Drug Therapy. Anal. Lett..

[58] El Manouni El Hassani S., Soers R.J., Berkhout D.J.C., Niemarkt H.J., Weda H., Nijsen T., Benninga M.A., De Boer N.K.H., De Meij T.G.J., Knobel H.H. (2020). Optimized Sample Preparation for Fecal Volatile Organic Compound Analysis by Gas Chromatography–Mass Spectrometry. Metabolomics.

[59] Tian Z., Zhuang X., Luo M., Yin W., Xiong L. (2020). The Propionic Acid and Butyric Acid in Serum but Not in Feces Are Increased in Patients with Diarrhea-Predominant Irritable Bowel Syndrome. BMC Gastroenterol..

[60] Eberhart B.L., Wilson A.S., O’Keefe S.J.D., Ramaboli M.C., Nesengani L.T. (2021). A Simplified Method for the Quantitation of Short-Chain Fatty Acids in Human Stool. Anal. Biochem..

[61] Watanabe D., Murakami H., Ohno H., Tanisawa K., Konishi K., Todoroki-Mori K., Tsunematsu Y., Sato M., Ogata Y., Miyoshi N. (2021). Stool Pattern Is Associated with Not only the Prevalence of Tumorigenic Bacteria Isolated from Fecal Matter but also Plasma and Fecal Fatty Acids in Healthy Japanese Adults. BMC Microbiol..

[62] van der Lelie D., Oka A., Taghavi S., Umeno J., Fan T.-J., Merrell K.E., Watson S.D., Ouellette L., Liu B., Awoniyi M. (2021). Rationally Designed Bacterial Consortia to Treat Chronic Immune-Mediated Colitis and Restore Intestinal Homeostasis. Nat. Commun..

[63] Gu H., Jasbi P., Patterson J., Jin Y. (2021). Enhanced Detection of Short-Chain Fatty Acids Using Gas Chromatography Mass Spectrometry. Curr. Protoc..

[64] Gray J., Guo B., Bearden R., Manka J. (2022). A Fast, Fully Validated GC-MS Method Using a Simplified Pretreatment for the Quantification of Short and Branched Chain Fatty Acids in Human Stool. J. Mass. Spectrom..

[65] Rahman M.N., Diantini A., Fattah M., Barliana M.I., Wijaya A. (2021). A Highly Sensitive, Simple, and Fast Gas Chromatography–Mass Spectrometry Method for the Quantification of Serum Short-Chain Fatty Acids and Their Potential Features in Central Obesity. Anal. Bioanal. Chem..

[66] Rohde J.K., Fuh M.M., Evangelakos I., Pauly M.J., Schaltenberg N., Siracusa F., Gagliani N., Tödter K., Heeren J., Worthmann A. (2022). A Gas Chromatography Mass Spectrometry-Based Method for the Quantification of Short Chain Fatty Acids. Metabolites.

[67] Kim K.-S., Lee Y., Chae W., Cho J.-Y. (2022). An Improved Method to Quantify Short-Chain Fatty Acids in Biological Samples Using Gas Chromatography–Mass Spectrometry. Metabolites.

[68] Li L., Chen L., Yang Y., Wang J., Guo L., An J., Ma X., Lu W., Xiao Y., Wang X. (2022). Characteristics of Gut Microbiome and Its Metabolites, Short-Chain Fatty Acids, in Children With Idiopathic Short Stature. Front. Endocrinol..

[69] Ramos-Garcia V., Ten-Doménech I., Moreno-Giménez A., Campos-Berga L., Parra-Llorca A., Solaz-García Á., Lara-Cantón I., Pinilla-Gonzalez A., Gormaz M., Vento M. (2022). GC-MS Analysis of Short Chain Fatty Acids and Branched Chain Amino Acids in Urine and Faeces Samples from Newborns and Lactating Mothers. Clin. Chim. Acta.

[70] Solar I., Ribeiro F.B., Barbosa M.G., De Oliveira Nascimento Freitas R.G.B., Hanada A.S., De Oliveira Ramos C., Sant’Ana M.R., Candreva T., De Almeida-Pititto B., Tura A. (2023). Short-Chain Fatty Acids Are Associated with Adiposity, Energy and Glucose Homeostasis among Different Metabolic Phenotypes in the Nutritionists’ Health Study. Endocrine.

[71] Xu J.J., Meng Y.T., Zou W.B., Zhao J.L., Fang X., Zhang Y., Zhou W., Zhang L., Wang K.X., Hu L.H. (2023). Cross-Sectional Evaluation of Gut Microbial–Host Cometabolites in Patients with Chronic Pancreatitis. J. Dig. Dis..

[72] Kang S., Yun J., Park H.-Y., Lee J.-E. (2023). Analytical Factors for Eight Short-Chain Fatty Acid Analyses in Mouse Feces through Headspace Solid-Phase Microextraction–Triple Quadrupole Gas Chromatography Tandem Mass Spectrometry. Anal. Bioanal. Chem..

[73] Wang Y., Liu H., Dong F., Xiao Y., Xiao F., Ge T., Li D., Yu G., Zhang T. (2023). Altered Gut Microbiota and Microbial Metabolism in Children with Hepatic Glycogen Storage Disease: A Case-Control Study. Transl. Pediatr..

[74] Kim Y., Kim G.-T., Kang J. (2023). Microbial Composition and Stool Short Chain Fatty Acid Levels in Fibromyalgia. Int. J. Environ. Res. Public Health.

[75] Thandar M., Yang X., Zhu Y., Zhang X., Chen Z., Huang S., Chi P. (2024). Dysbiosis of Gut Microbiota and Metabolites Is Associated with Radiation-Induced Colorectal Fibrosis and Is Restored by Adipose-Derived Mesenchymal Stem Cell Therapy. Life Sci..

[76] Xie T., Fan X., Pang H., Zang T., Wu N., Liu J., Li Z., Li S., Zhu Q., Slack J.E. (2024). Association between Gut Microbiota and Its Functional Metabolites with Prenatal Depression in Women. Neurobiol. Stress.

[77] Kulecka M., Czarnowski P., Bałabas A., Turkot M., Kruczkowska-Tarantowicz K., Żeber-Lubecka N., Dąbrowska M., Paszkiewicz-Kozik E., Walewski J., Ługowska I. (2024). Microbial and Metabolic Gut Profiling across Seven Malignancies Identifies Fecal Faecalibacillus Intestinalis and Formic Acid as Commonly Altered in Cancer Patients. Int. J. Mol. Sci..

[78] Czarnowski P., Bałabas A., Kułaga Z., Kulecka M., Goryca K., Pyśniak K., Unrug-Bielawska K., Kluska A., Bagińska-Drabiuk K., Głowienka-Stodolak M. (2024). Effects of Soluble Dextrin Fiber from Potato Starch on Body Weight and Associated Gut Dysbiosis Are Evident in Western Diet-Fed Mice but Not in Overweight/Obese Children. Nutrients.

[79] Allen J.M., Mailing L.J., Niemiro G.M., Moore R., Cook M.D., White B.A., Holscher H.D., Woods J.A. (2018). Exercise Alters Gut Microbiota Composition and Function in Lean and Obese Humans. Med. Sci. Sports Exerc..

